# Left-Sided Pleural Effusion as a Complication Following Splenic Embolisation: A Case Report and Literature Review

**DOI:** 10.7759/cureus.96778

**Published:** 2025-11-13

**Authors:** Kabyar Cho, Chern Lee Choy, Shivani Upadhyay, Tasaduksultan Khan

**Affiliations:** 1 Intensive Care Unit, Furness General Hospital, University Hospitals of Morecambe Bay NHS Foundation Trust, Barrow-in-Furness, GBR; 2 Respiratory Medicine, Furness General Hospital, University Hospitals of Morecambe Bay NHS Foundation Trust, Barrow-in-Furness, GBR

**Keywords:** iatrogenic complication, intercostal chest tube drain, left sided pleural effusion, splenic artery embolisation, spontaneous splenic rupture

## Abstract

Most pleural effusions arise due to intrathoracic aetiologies. Left-sided pleural effusion can occur as a complication of splenic artery embolisation (SAE). Nowadays, it is standard practice to perform SAE as a nonoperative management for splenic injury in haemodynamically stable patients. Although it is far less invasive compared to surgical splenectomy, the procedure is associated with possible complications ranging from splenic atrophy and splenic infarction to pleural effusion, fever, and coil migrations. Mild to moderate pleural effusion can be managed conservatively with watchful monitoring, whereas refractory and symptomatic effusions would require repeated thoracocentesis or intercostal chest drain (ICD) insertion.

We report a case of a 59-year-old female patient presenting with shortness of breath, associated with pyrexia and raised inflammatory markers. She had undergone a proximal splenic artery embolisation (PSAE) for spontaneous splenic rupture one month prior to her current presentation. Computed tomography (CT) imaging revealed a large left-sided pleural effusion, which caused a contralateral mediastinal shift. Additionally, there was evidence of a splenic infarct post-embolisation and an associated peri-splenic collection. She was managed with an ICD insertion, which was removed after six days, and she was discharged home safely, with a residual loculated pleural effusion. A follow-up chest X-ray at six weeks showed that the remaining effusion had resolved completely without any further complications. Our case highlights the importance of recognising extrapulmonary causes of left-sided pleural effusions, especially in patients who have undergone SAE.

## Introduction

Pleural effusions are a common medical problem encountered in today’s medical practice. In a normal, healthy person, production and resorption of the pleural fluid by the pleura is regulated. Pathophysiological changes, such as low oncotic pressure, elevated pulmonary capillary pressure, lymphatic flow obstruction, and impaired negative intrapleural pressure, lead to disruption in this equilibrium, resulting in an accumulation of fluid in the pleural space [1-3]. Pleural effusions are further distinguished by type: either transudate or exudate. Infections, malignancy, and systemic diseases remain the most common underlying causes of exudative pleural effusion, whereas congestive cardiac failure, hepatic cirrhosis, and nephrotic syndromes manifest as a transudate [1]. Effusions related to structures beneath the diaphragm are less commonly suspected [4]. Currently, there is limited literature surrounding severe, complicated pleural effusion post-splenic artery embolisation (SAE). Management primarily depends on the size of the effusion and the clinical status of the patient. It is important to initially investigate the effusion, send it for appropriate diagnostic workup and imaging, and offer necessary interventions, such as chest drain insertion or further decortication [5]. We present a case of a large, left-sided pleural effusion with compressive atelectasis of the left upper and lower lobes, following SAE, in a 59-year-old female patient.

## Case presentation

A 59-year-old female patient presented to the Emergency Department with a two-day history of central abdominal pain, associated with non-bilious vomiting and generalised weakness. Three months prior to this presentation, she was involved in a motorbike incident, where she had fallen off the bike onto the platform, travelling at a speed of 25 to 30 miles per hour. She sustained no major injuries at the time and was discharged home with analgesia for her back pain. She denied any other traumatic causes. She had no other complaints: no chest pains, dyspnoea, fever, irregular bowel habits, or lower urinary tract symptoms. Earlier that month, she presented to the hospital for symptomatic bradycardia/sinus node dysfunction, which required her to have a cardiac permanent pacemaker (PPM) insertion. Following her PPM insertion, she was symptomatic with marked hypotension and was monitored in the intensive care unit, even though she did not require any inotropic support. With a troponin rise, accompanied by dynamic electrocardiogram (ECG) changes, she was suspected of acute coronary syndrome (ACS) and transferred to a tertiary centre for an urgent coronary angiogram. However, due to her echocardiogram findings of apical hypokinesia with impaired left ventricular systolic function (estimated at 45% to 50%) and coronary angiogram findings of minimal coronary artery disease, she was diagnosed with Takotsubo cardiomyopathy. Cardiology performed a repeat echocardiogram and confirmed the diagnosis of Takotsubo, with improvement in her left ventricular systolic function. Her other past medical history included monoclonal gammopathy, osteoarthritis, anterior resection for rectal cancer, bipolar affective disorder, depression, and hereditary glaucoma. She was not on any regular antiplatelet agents or anticoagulants prior to this episode. Her vital signs on arrival to the hospital were as follows: non-invasive blood pressure of 120/58, heart rate of 73, respiratory rate of 20, oxygen saturations of 97% on room air, and a temperature of 37°C. Abdominal examination findings included tenderness on deep palpation of her epigastric, left upper quadrant, and lumbar regions, with no features of peritonism. The haemoglobin count and infection markers on admission were relatively normal, as shown in Table 1. Her renal and liver profiles were unremarkable as well.

**Table 1:** Infection and inflammatory markers on admission.

Laboratory parameters	Values	Reference range
Haemoglobin (g/L)	121	115 - 165
Haematocrit (%)	36.2	36.0 - 46.0
Platelets (x10^9/L)	316	150 - 400
WBC count (x10^9/L)	9.8	4.0 - 10.0
Neutrophil count (x10^9/L)	7.7	2.0 - 7.5
C-reactive protein (mg/L)	12.7	0.0 - 5.0

**Figure 1:**
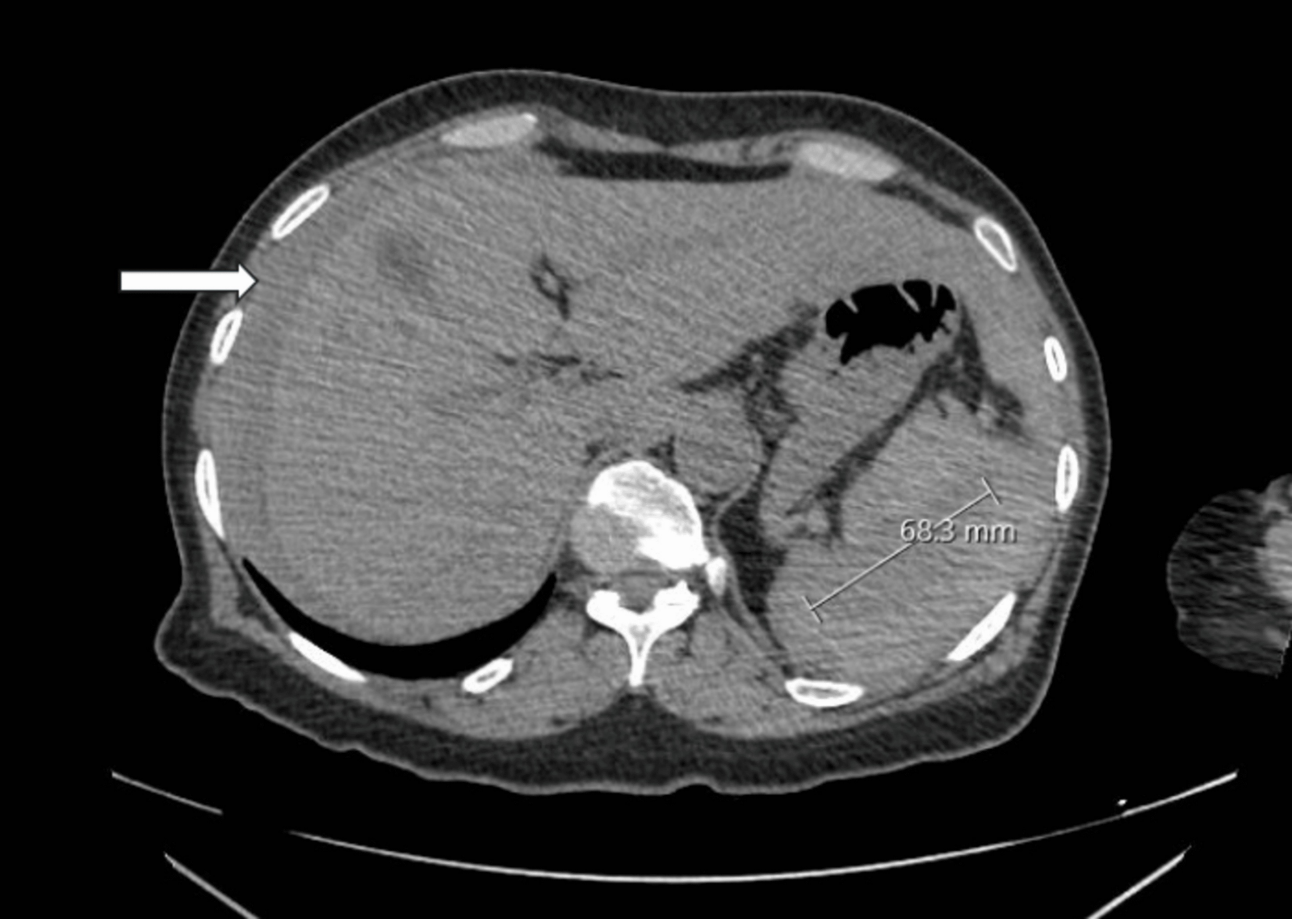
Computed tomography of abdomen and pelvis with contrast demonstrating splenomegaly (measuring 68.3 mm) due to intraparenchymal haematoma. The white arrow indicates haemoperitoneum around the liver.

Her venous blood gas indicated a metabolic alkalosis due to hyperventilation, with a pH of 7.519, pCO_2_ 4.08 kPa, base excess 2.6 mmol/L, and bicarbonate level 24.9 mmol/L, as tabulated in Table 2. Due to her presentation and a raised lactate of 2.8 mmol/L, an urgent computed tomography abdomen and pelvis (CT AP) with contrast was performed, which showed an actively bleeding splenic rupture with extensive haemoperitoneum (Figure 1). She was promptly transferred to a tertiary centre for ultrasound-guided embolisation of the spleen. The interventional radiologist performed a proximal splenic artery embolisation (PSAE), deploying four microcoils in the main splenic artery, which successfully occluded the vessel and controlled the bleeding. 

**Table 2:** Venous blood gas results on admission.

Laboratory parameters	Values	Reference range
pH	7.519	7.35 - 7.45
pCO_2_ (kPa)	4.08	4.7 - 6.0
HCO_3_^- ^(mmol/L)	24.9	22.0 - 26.0
Base excess (mmol/L)	2.6	-2.0 - +2.0
Lactate (mmol/L)	2.8	0.5 - 2.2

**Figure 2:**
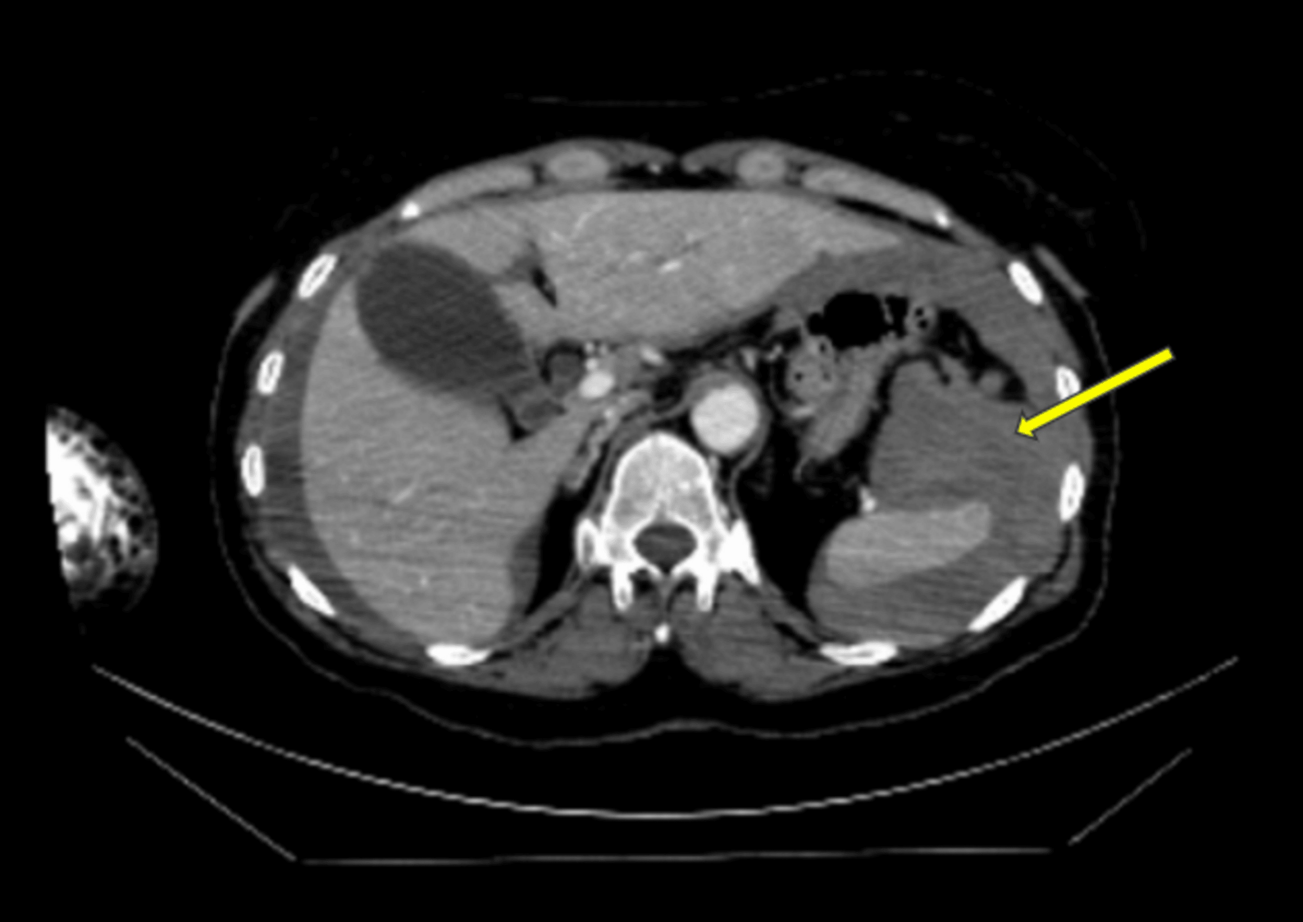
Computed tomography angiogram of the abdomen at the splenic level. The yellow arrow demonstrates an enlarged spleen with surrounding hypoattenuation, which indicates areas of infarction post splenic artery embolisation.

Two days later, she sustained a drop in haemoglobin from 96 to 64 g/L and necessitated a blood transfusion. A CT angiogram of her abdomen showed an enlarged spleen with heterogeneous areas of hypodensity, indicating infarction, which appearance was expected post-embolisation (Figure 2). The rest of the study was normal; there was no other active contrast extravasation seen to suggest a recurrent bleed. About five weeks later, she was repatriated to our district hospital for rehabilitation and ongoing pain management. 

During her rehabilitation period at our hospital, she started having a temperature of 38°C on multiple occasions and became more tachypnoeic, with dyspnoea at rest. Her inflammatory markers were raised, with values demonstrating an upward trend over the days preceding symptom onset (Table 3).

**Table 3:** Trend of inflammatory markers and haemoglobin over the period of symptom onset.

Laboratory parameters	Day 1	Day 2	Day 3	Day 4	Day 5	Reference range
Haemoglobin (g/L)	113	102	102	91	89	115 - 165
Haematocrit (%)	33.7	30.3	31.2	26.4	26.3	36 - 46
Mean cell volume (fL)	87.9	86.8	85.4	85.6	85.5	77 - 100
WBC count (x10^9/L)	8.8	9.3	9.9	8.9	8	4 - 10
Neutrophil count (x10^9/L)	6.3	6.7	7.1	6.3	5.3	2.0 - 7.5
C-reactive protein (mg/L)	69.8	154.7	198.6	165.3	131.7	0.0 - 5.0

She was reviewed by the out-of-hours medical team, and a CT AP with contrast was requested urgently to assess for any complications post-interventional radiology (IR) embolisation: increasing peri-splenic collection, abscess formation, new bleed, or other intra-abdominal pathologies.

**Figure 3:**
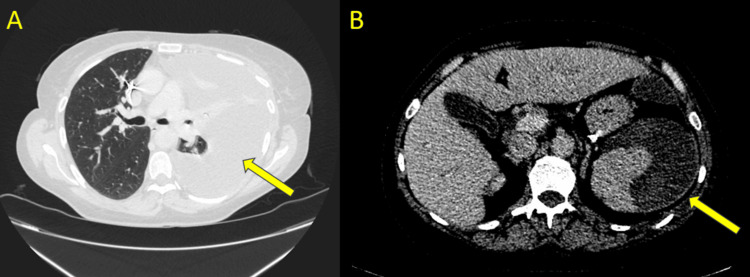
Computed tomography abdomen and pelvis at the level of the bases of the lungs (A) and the spleen (B). The yellow arrow in slide (A) demonstrates a left-sided pleural effusion. The yellow arrow in slide (B) demonstrates a well-encapsulated peri-splenic haematoma, which appears more organised compared to imaging on admission.

**Figure 4:**
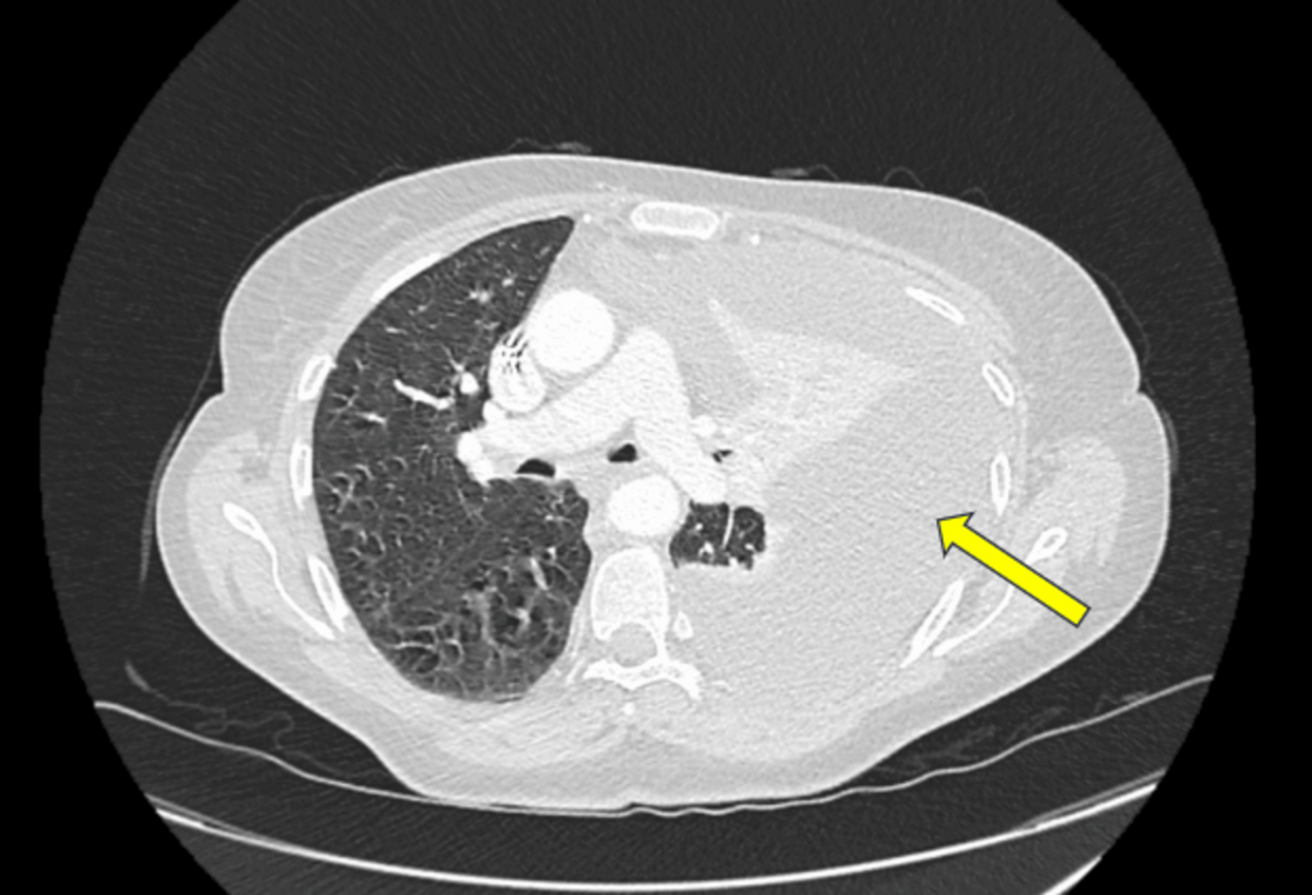
Computed tomography of thorax with contrast demonstrating a large pleural effusion in the left lung. The yellow arrow annotates a large left-sided pleural effusion, which has caused a mediastinal shift.

The CT scan showed a left pleural effusion with a well-encapsulated peri-splenic haematoma, appearing smaller in volume (Figure 3). After consultation with the respiratory team, a decision was made to request a contrast CT thorax to further delineate the nature and extent of the pleural effusion. The CT thorax showed a large left-sided pleural effusion, causing mediastinal shift, alongside compressive atelectasis of the left upper and lower lobes, with partial aeration of the left apical posterior segment (Figure 4). The radiological analysis of the effusion concluded that the density of the fluid was unlikely to represent a haemothorax.

**Figure 5:**
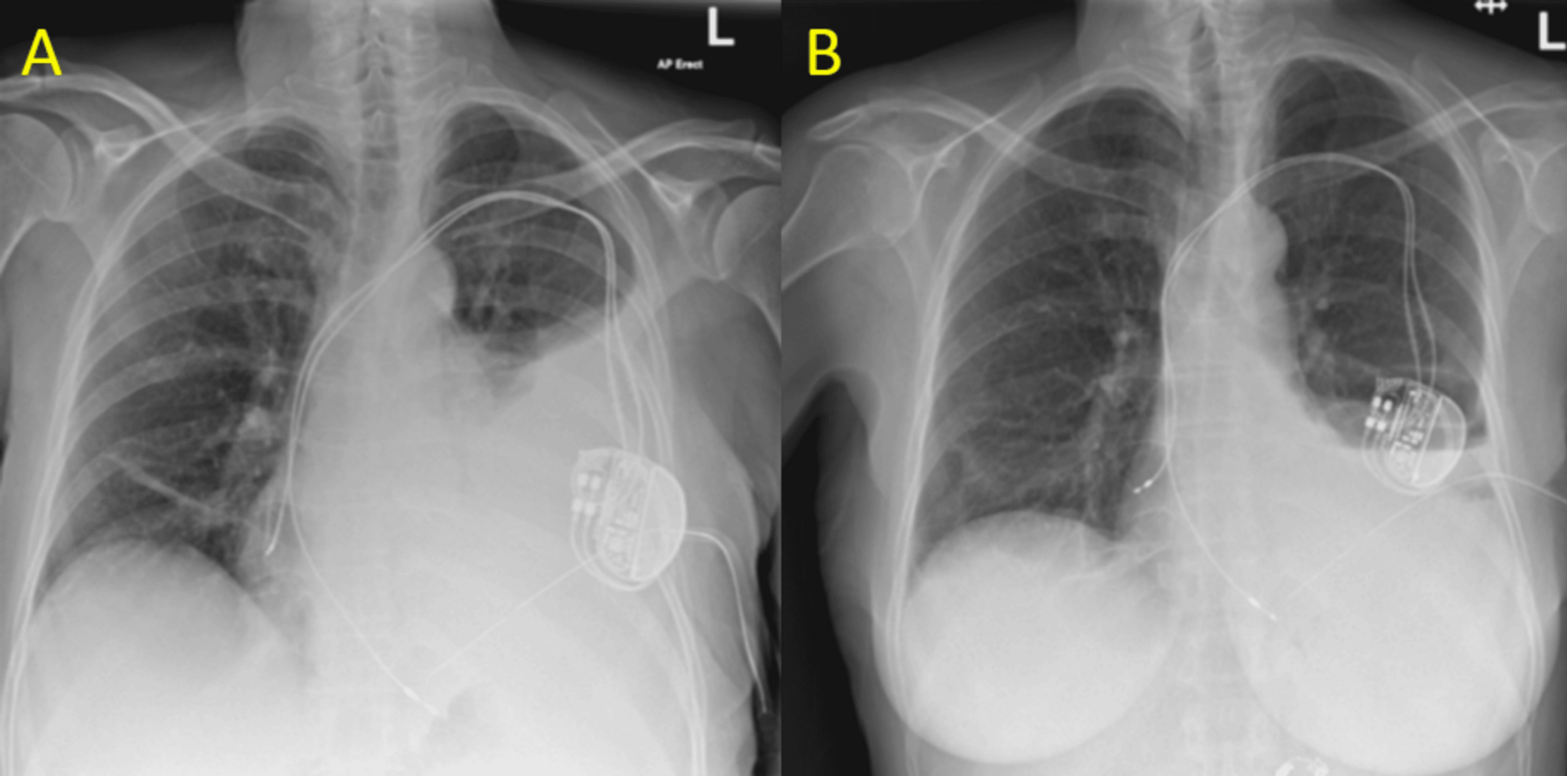
Chest X-rays. (A) Chest X-ray showing a left-sided pleural effusion upon intercostal chest drain insertion. (B) Chest X-ray on day 6 showing a remaining loculated effusion at the base of the left lung with the intercostal chest drain in situ.

Following this, the respiratory team inserted a Seldinger intercostal chest drain (ICD), draining a total of 1200 mL of serosanguinous fluid. The pleural fluid analysis was consistent with an exudative effusion, with a pH of 7.37, protein level of 37 g/L, glucose level of 5.4 mmol/L, and lactate dehydrogenase (LDH) level of 930 IU/L. Bacterial and fungal cultures were negative. Histology results showed mainly lymphocytic effusion, with no malignant cells seen. Six days later, there was minimal output from her drain despite increasing suction pressure. Figures 5A-5B compare the chest X-ray on the day of ICD insertion with that on day 6, demonstrating a persistent loculated effusion that was not amenable to drainage.

**Figure 6:**
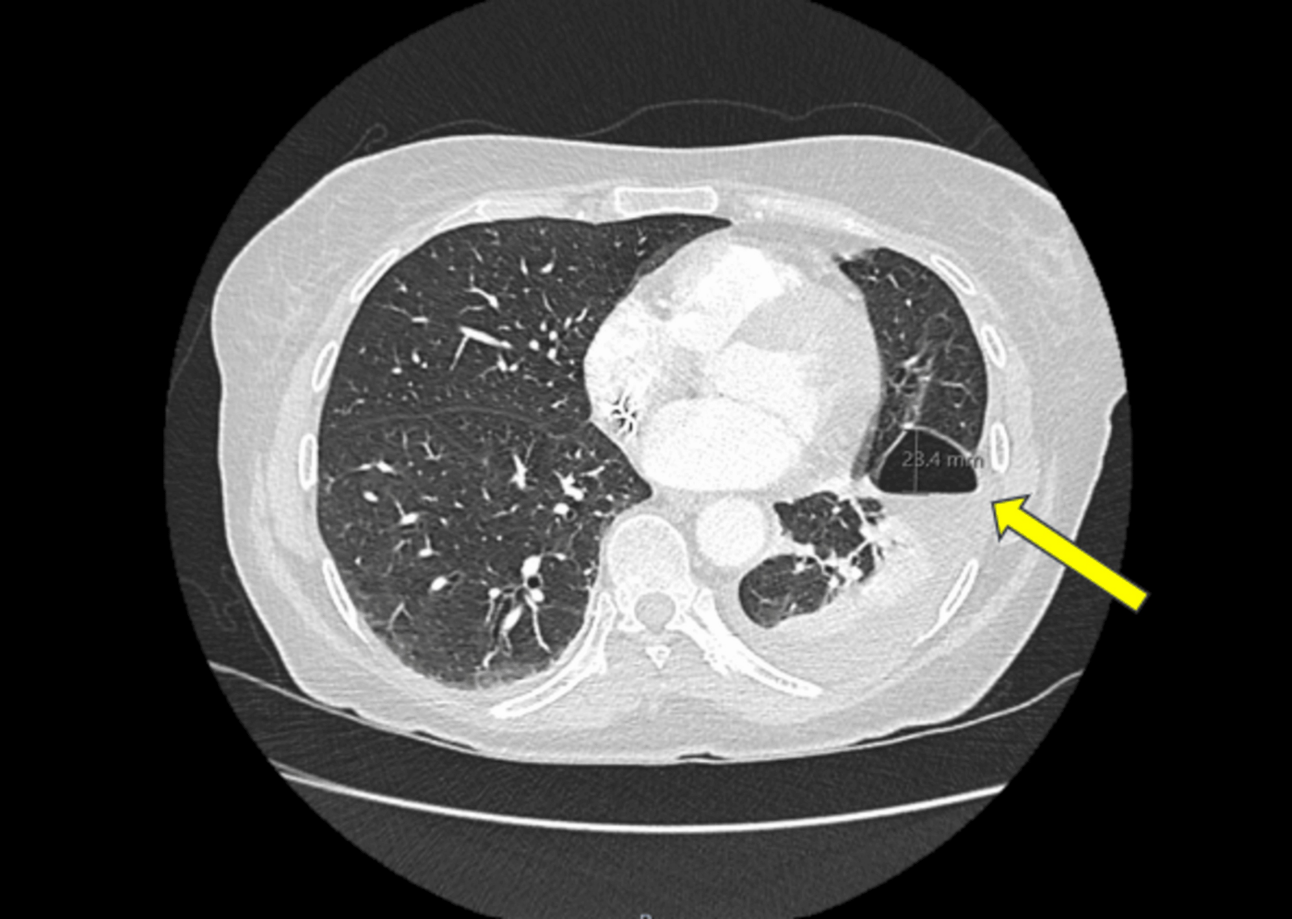
Computed tomography pulmonary angiogram demonstrating a residual mild left pleural effusion with an air-containing pocket, measuring 23.4 mm (yellow arrow), seen posterior to the left lung oblique fissure.

The medical team decided to remove her chest drain due to a higher chance of self-resorption and discharged her home with safety-netting advice and follow-up imaging in four to six weeks' time. Four weeks later, she presented to the hospital again with pleuritic chest pain and right upper quadrant pain. Due to her recent admission history and a moderate Wells’ score for pulmonary embolism, a CT pulmonary angiogram (CT PA) was performed. She did not sustain any venous thromboembolism but did show a residual mild left pleural effusion with an air-containing pocket seen posterior to the left lung oblique fissure, as expected post-drain removal (Figure 6). She was treated conservatively for her somatic pain and discharged to the community pain team. 

**Figure 7:**
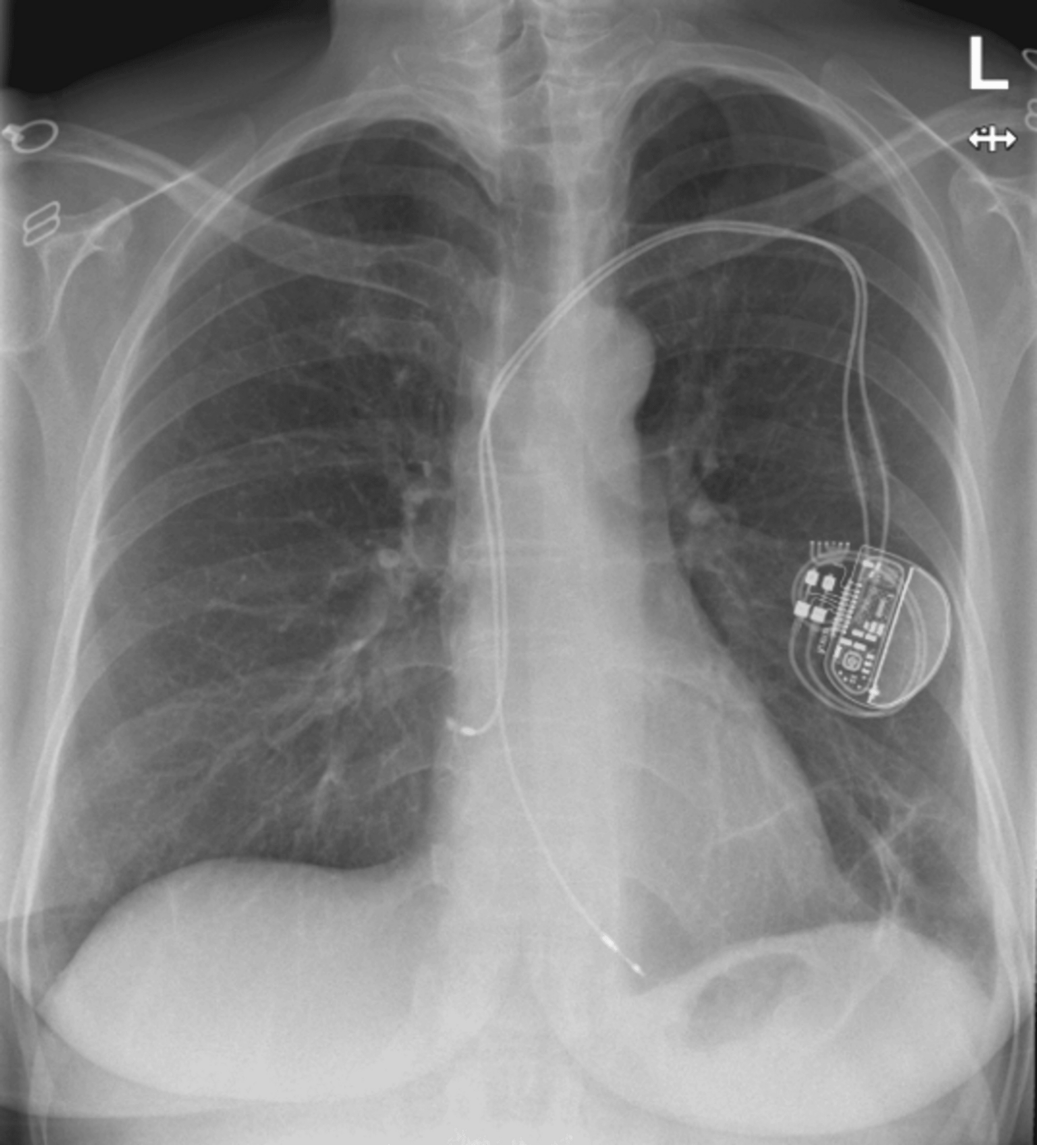
Chest X-ray showing complete resolution of the left-sided pleural effusion, approximately six weeks post-chest drain removal.

A follow-up chest X-ray six weeks later showed near-complete resolution of the left pleural effusion, with minimal subsegmental atelectasis in the lower zone of the left lung field (Figure 7). Based on her significantly improved clinical status and chest X-ray results, she was discharged from both the respiratory and surgical teams. A follow-up contrast CT AP was done 10 days later, which showed interval partial resolution of the peri-splenic collection, measuring approximately 80 × 35 mm compared to its previous measurement of 100 × 65 mm, with no new acute features. 

## Discussion

Pleural effusion is an abnormal, excessive accumulation of fluid in the pleural cavity [1]. It is commonly encountered in today’s clinical practice, with an estimated 1.5 million cases in the United States and 200000 to 250000 in the United Kingdom every year [5]. The volume of fluid is determined by the balance between the hydrostatic pressure and plasma oncotic pressure differences in the systemic capillary system [2]. Pleural fluid is resorbed via lymphatic vessels in the parietal pleura. Factors such as low oncotic pressure in a state of hypoalbuminaemia and high pulmonary capillary pressure in congestive cardiac failure can cause a shift in this equilibrium, resulting in pathophysiological collection of pleural fluid. Other causes include increased permeability into the space through the porous diaphragm, negative intrapleural pressure, and impaired lymphatic drainage [1]. 

Pleural effusion is usually classified as transudative or exudative, based on its biochemical characteristics, with Light’s criteria being the most widely used method for this distinction [6]. The most common causes of transudative effusion, which are often bilateral, include congestive heart failure, hepatic hydrothorax, nephrotic syndrome, and, less commonly, myxoedema and sarcoidosis [1,5]. Exudative effusions can result from inflammatory or infectious causes. These include parapneumonic effusion due to infection (bacterial, tuberculous, viral, or fungal), pulmonary infarction, neoplasms, malignancy, and systemic inflammatory conditions such as rheumatoid arthritis [1,5,6]. It is crucial to understand the type of effusion to determine further diagnostic workup and management. 

When subdiaphragmatic structures lead to pleural effusions, the diagnostic approach is often considered more challenging. These presentations are less prevalent and may manifest with non-specific and subtle abdominal signs, which can conceal the underlying pathology [7]. Pleural effusion can result from hepatic and subphrenic abscesses, gastrointestinal-pleural fistulas, and pancreatic aetiologies. Subdiaphragmatic organ inflammation can lead to impaired lymphatic drainage from the pleural cavity and increased permeability of pleural capillaries, resulting in a reactive lymphocytic pleural effusion [4,7]. Pleural effusions arising due to splenic infarction as a complication of SAE are less frequently suspected, and diagnosis can be delayed if clinicians focus their assessment primarily on pulmonary and cardiac causes [8]. 

Prior to her SAE, there were no identifiable causes that could have led to her splenic rupture, apart from her history of a low-grade back injury, which she sustained following a motor vehicle accident three months earlier. Although a splenic bleed or rupture typically occurs almost immediately after blunt abdominal trauma, there remains a possibility of delayed presentation. Delayed splenic rupture (DSR) was first described in 1907 by Baudet as occurring 48 hours or later following an initial injury to the spleen [9]. Delayed rupture is rare, and most cases are reported to occur within a week of the injury, although it can present up to 70 days after minor trauma [9,10]. A report by Basukala et al. (2021) highlighted the importance of this phenomenon, even following trivial abdominal trauma, where a patient accidentally bumped into furniture at home [11]. Given the nature of the injury in our patient and the timeframe, a direct causal relationship remains speculative, although it cannot be entirely excluded. 

The sequence of cardiac and splenic events in this patient was unusual. Further investigations were performed to determine any underlying systemic conditions or lymphoproliferative disorders. A full multiple myeloma screen was performed, which returned negative. Serum free light chain analysis showed kappa and lambda levels within normal limits, with a normal kappa/lambda ratio. Serum protein electrophoresis identified a small paraprotein band, as expected from her monoclonal gammopathy. Other investigations, including inflammatory markers, LDH, immunoglobulin levels, and β2-microglobulin levels, were unremarkable. In addition, her skeletal survey X-ray did not detect any lytic lesions, and these findings made the splenic bleed unlikely to be the result of an underlying haematological or systemic condition. 

Whilst haematological malignancies are thought to be the most common causes of atraumatic splenic rupture, other infectious causes, such as viral, protozoan, and parasitic infections, are responsible for a small percentage of splenomegaly and spontaneous splenic rupture [12]. Our patient's travel history was insignificant, and her non-invasive liver screen was negative. Although Epstein-Barr virus (EBV) and cytomegalovirus (CMV) serologies were not performed at the time, her clinical presentation and past medical history did not raise immediate suspicion for these viral infections. A systematic review by Renzulli et al. (2009) identified 845 splenic ruptures between 1980 and 2008; 7% were found to be atraumatic-idiopathic, and 93% atraumatic-pathological [13]. It is often underdiagnosed due to the absence of obvious trauma or an underlying splenic disease. The typical presentation of splenic rupture involves tenderness and guarding in the left upper quadrant. Our patient presented to the hospital with epigastric pain and minimal left upper quadrant tenderness, associated with non-bilious vomiting. There were no signs of guarding or rigidity on examination. Given the findings of splenic rupture and extensive haemoperitoneum on CT scan, she underwent IR embolisation in a timely manner. 

Currently, the American Association for the Surgery of Trauma (AAST) splenic injury scale is used to grade the degree of splenic trauma and guide management. Patients who are haemodynamically unstable, with a major hilar vascular injury devascularising more than 25% of the spleen, may require urgent laparotomy and splenectomy, whereas Grade I to III injuries involving capsular and subcapsular tears can be managed conservatively. SAE by IR remains an alternative option for haemodynamically stable candidates with high-grade injuries, proving useful for preserving splenic function [14]. The use of SAE for nonoperative salvage of patients with splenic trauma was first described by Sclafani et al. in 1995 [15]. 

PSAE is recommended in cases of multifocal injuries or when no vascular angiographic disturbances are identified, whereas distal splenic artery embolisation (DSAE) is reserved for focal vascular injuries. PSAE reduces perfusion pressure, leading to increased haemostasis and healing of the spleen [16]. SAE carries its own complications, which can be categorised as major or minor. Major complications include splenic abscess, infarction, cysts, and contrast-induced renal insufficiency, while minor complications include coil migration, fever, and left-sided pleural effusions [17-19]. In a large case series of 599 patients, Alomar et al. observed left-sided pleural effusions in 13.1% of SAE cases, underscoring that this represents a relatively common minor complication [19]. Due to its relatively small size and self-limiting nature, left-sided pleural effusion following SAE is treated conservatively in most cases. Recent studies have also shown no significant difference in complication rates between PSAE and DSAE [20]. Although uncommon, splenic infarction and severe left-sided pleural effusion can occur after SAE, as seen in our patient. Patients with clinically significant pleural effusions usually require thoracocentesis to aid recovery. In the case of DSAE, several studies have suggested that the middle and lower lobes of the spleen are more favourable sites for embolisation, as the upper lobe is associated with an increased risk of pleural effusion [21,22]. There is limited evidence on symptomatic pleural effusion requiring thoracocentesis in patients who have undergone PSAE. Additionally, the timing of pleural effusion following SAE is rarely described in the literature. Pandey et al. reported a case in which the effusion occurred three days after the procedure, in contrast to our patient, who presented four weeks post-embolisation [8]. 

The pathophysiology underlying pleural effusion in the context of SAE is not well understood. There could be several factors contributing to the development of a pleural effusion. A study by Warren and Gibbons (1991) postulated that the exudative fluid accumulation in the pleural cavity is likely due to the enlarged and infarcted spleen compressing the posterior lymphatic drainage system, thereby impairing lymphatic drainage from the pleural cavity. Additionally, subphrenic irritation caused by the perisplenic haematoma increases capillary permeability in the pleural cavity, leading to a reactive exudative pleural effusion [23,24]. The SAE procedure itself could directly irritate the spleen, resulting in increased splenic inflammation and promoting permeability of the splenic vessels. This, in turn, leads to a shift of haemorrhagic fluid into the pleural space through the porous diaphragm, often resulting in a left-sided pleural effusion [8,24]. These are the mechanisms considered to result in an exudative pleural effusion in our patient. 

There is currently no standardised management for pleural effusion following SAE. The management approach should be individualised, after thorough assessment of presenting signs and symptoms in each patient [24]. It is important for clinicians to be aware of extrapulmonary causes of pleural effusions and to consider necessary abdominal imaging promptly to evaluate any abdominal pathologies or procedures leading to pleural effusion. Surgeons and IR clinicians should also familiarise themselves with complications post-SAE, and anticipate symptomatic pleural effusion in such patients. As these effusions carry a risk of recurrence, meticulous monitoring and regular follow-up imaging in these patients may be required [8].

## Conclusions

In conclusion, this is a case of left-sided pleural effusion due to splenic infarction post-SAE, in a patient who sustained a spontaneous splenic rupture. Our case highlights the importance of including extrapulmonary causes in the diagnosis of left-sided pleural effusions. Clinicians should keep their differentials broad and request appropriate investigations and imaging promptly to facilitate a timely diagnosis. Early identification of uncommon causes, such as splenic pathology, can significantly influence management decisions. Emphasis on thorough history-taking, particularly regarding preceding trauma or intra-abdominal interventions, remains imperative to make an accurate diagnosis and guide further management.
